# Supplementary material for: MbWRKY53, a M. baccata WRKY Transcription Factor, Contributes to Cold and Drought Stress Tolerance in Transgenic Arabidopsis thaliana
Source: Int J Mol Sci. 2024 Jul 11;25(14):7626. doi: 10.3390/ijms25147626 (PMC11276640; doi:10.3390/ijms25147626)
Supplement: Supplementary file 1 [file ijms-25-07626-s001.zip › ijms-3061395-supplementary.pdf]

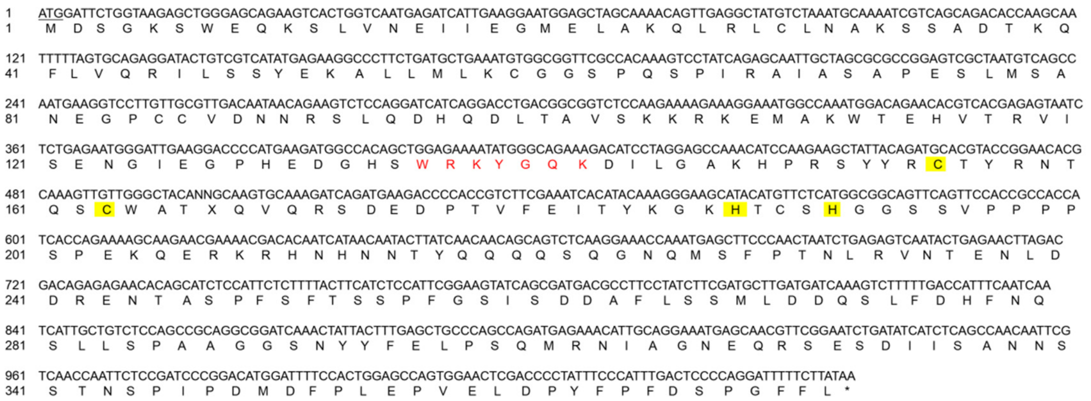

Figure S1: Gene sequences and aa sequences of *MbWRKY53*. The black underline represents the start codon and the stop codon; the conserved amino acid sequence unique to WRKY is shown in red; the yellow parts are the C2H2 zinc finger motif of WRKY.

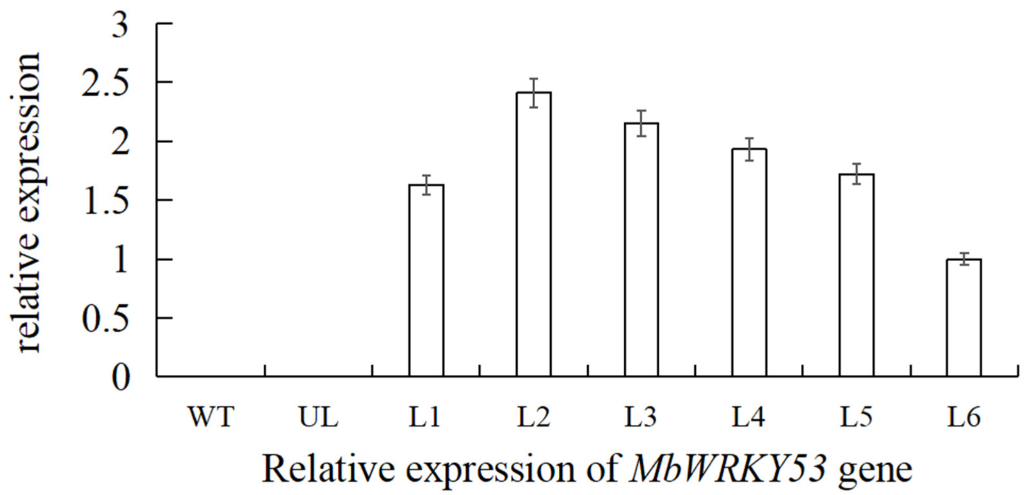

Figure S2: Relative expression levels of *MbWRKY53* gene in transgenic *Arabidopsis*. L1-L6 were the 6 selected transgenic lines.

Table S1 Primers used in this study.

Primer	Sequence (5'-3')
MbWRKY53-F	ATGGATTCTGGTAAGAGCTGG
MbWRKY53-R	TTATAAGAAAAATCCTGGGG
MbWRKY53-2F	GAGCTCGGTACCCGGGGATCCATGGATTCTGGTAAGAGCTGG
MbWRKY53-2R	TCTAGAGGATCCCCGGGTACCTTTATAAGAAAAATCCTGGGG
MbWRKY53-qF	TGTGAGATTAGGGATGAAA
MbWRKY53-qR	TTTCATCCCTAATCTCACA
AtSOS1-F	CTGCTTGCTACATTTCTGC
AtSOS1-R	TGCTTCCTCTCCTTCCTT
AtDREB2A-F	TGGAGAATGGTGCGGAAGA
AtDREB2A-R	CTCCACTCTGATCATAAACTGCCAT

AtRAB18-F	GCTTGCGATTTGGGATACAG
AtRAB18-R	CCAGACGAACCTTCAGCAGT
AtP5CS1-F	GATACGGATATGGCAAAGCG
AtP5CS1-R	CCAAGTCCAAATCGGAAACC
AtCOR6.6-F	ACAGGCGGGAAAGAGTAT
AtCOR6.6-R	TGGAAGGCATTCTTGTTG
AtRD29b-F	CAACGAGGGGAAGATAAAAGTGT
AtRD29b-R	AGCCAGATGATTTTGGAGCCT

Table S2 The PCR reaction system.

Components	Volume
cDNA	1.5 μL
MbWRKY75-F	1.0μL
MbWRKY75-R	1.0 μL
2×Easy Taq® PCR SuperMix	12.5 μL
dd H_2O	9.0 μL
Total Volume	25 μL

Table S3 PCR reaction conditions.

Step	Temperature	Time
Pre-denaturation	97℃	5 s
Denaturation	97℃	5 s
Anneal	55-65℃	10 s
Extend	72℃	20 s

Table S4 The RT-PCR reaction system.

Component	Volume
Primer-qF	2.0 μL
Primer-qR	2.0 μL
5×BlazeTaq qPCR Mix[a]	4.0 μL
cDNA	2.0 μL
dd H2O	10.0μL
Total Volume	20.0 μL

Table S5 RT-qPCR reaction conditions.

Step	Temperature	Time
Pre-denaturation	95°C	30 s
Denaturation	72°C	10 s
Annealing and elongation	55°C	30 s
